# Human migrations, anthropogenic changes, and insect-borne diseases in Latin America

**DOI:** 10.1186/s13071-024-06598-7

**Published:** 2025-01-09

**Authors:** André B. B. Wilke, Priscilla Farina, Marco Ajelli, Angelo Canale, Filipe Dantas-Torres, Domenico Otranto, Giovanni Benelli

**Affiliations:** 1 Laboratory for Computational Epidemiology and Public Health, Department of Epidemiology and Biostatistics, Indiana University School of Public Health, Bloomington, IN USA; 2 Department of Agriculture, Food and Environment, University of Pisa, Pisa, Italy; 3 Department of Immunology, Aggeu Magalhães Institute, Oswaldo Cruz Foundation (Fiocruz), Recife, Brazil; 4 Department of Veterinary Medicine, University of Bari, Valenzano, Italy; 5 Department of Veterinary Clinical Sciences, City University of Hong Kong, Hong Kong, China

**Keywords:** Arbovirus, Chagas disease, Chikungunya, Dengue, Epidemiology, Epidemics, Leishmaniasis, Malaria, Migration, Mosquito, Trypanosomiasis, Yellow fever, Zika virus

## Abstract

Rapid urbanization and migration in Latin America have intensified exposure to insect-borne diseases. Malaria, Chagas disease, yellow fever, and leishmaniasis have historically afflicted the region, while dengue, chikungunya, and Zika have been described and expanded more recently. The increased presence of synanthropic vector species and spread into previously unaffected areas due to urbanization and climate warming have intensified pathogen transmission risks. This review examines recent outbreaks and reemergence of insect-borne diseases through five case studies: (i) malaria transmission linked to political instability and large-scale migration through the Amazon jungle; (ii) the expansion of triatomine bug habitats into overcrowded, substandard urban settlements, increasing Chagas disease incidence; (iii) the influence of movement and ecotourism in the Amazonia on yellow fever transmission in peri-urban areas; (iv) the spread of visceral leishmaniasis driven by deforestation and human–canine movement; and (v) dengue outbreaks in rural Amazon regions, spurred by urbanization and rural development. The findings underscore the complex interactions among vectors, pathogens, and shifting environmental and social conditions, complicating predictability and control. Addressing the social, economic, and political determinants of health is crucial to reducing disease transmission. Key measures include scaling vaccine coverage, especially for dengue and yellow fever; developing vaccines and treatments for neglected diseases; improving housing and sanitation; strengthening vector surveillance and control; fostering community engagement; enhancing data-driven interventions.

**Graphical Abstract:**

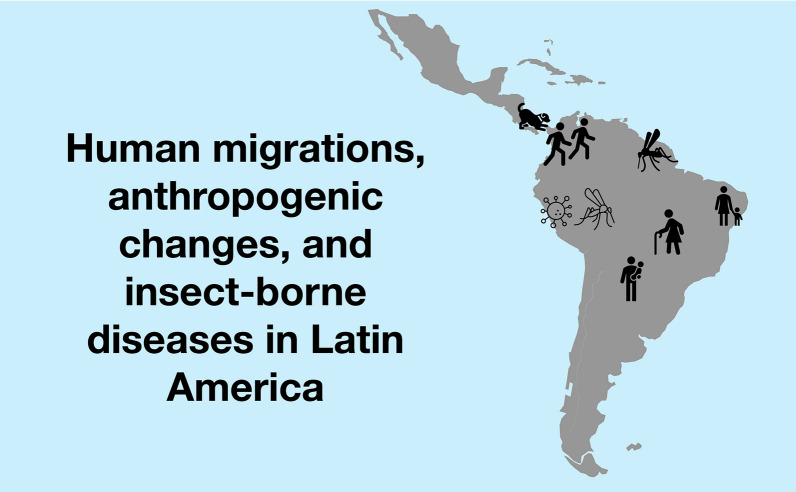

## Insect-borne diseases and anthropogenic changes: what is going on today?

Human migrations and anthropogenic changes in the environment are major drivers for the community composition, abundance, and richness of mosquito species [1]. Deforestation, agricultural development, land-use, and urbanization processes generally result in a decrease in insect diversity, followed by a surge in the abundance of those species that are better suited to urban life [2]. For instance, this is the case of the mosquitoes *Aedes albopictus* (Skuse) and *Aedes aegypti* (L.) (Diptera: Culicidae) and some phlebotomine sand flies (Diptera: Psychodidae: Phlebotominae) [3–5].

Insect-borne disease transmission is a complex ecological phenomenon where multiple species (i.e., vectors, hosts, reservoir species, dead-end hosts, and others) are involved in the transmission cycle. For instance, the higher abundance of mosquitoes and the elevated human population density increase the exposure level to these vectors and the risk of mosquito-borne pathogen transmission in urban as compared to rural areas [6]. In urban settlements, diseases such as dengue are linked to insufficient water supplies for personal and domestic hygiene and appropriate solid waste disposal measures [7]. Furthermore, given that urbanization is on the rise in Latin America, it is crucial to understand the impact of biodiversity loss and the increasing presence of synanthropic insects on the incidence of insect-borne diseases [8]. Since the 1950s, Latin America has experienced a significant boom in terms of urbanization, with an increased flow of people migrating from rural areas to cities in search of employment and improved socioeconomic opportunities. Such complex migration processes are dynamic and can be influenced by numerous economic, political, social, and environmental factors [9]. Rural development and deforestation policies have historically had an impact on migration waves in Latin America [9]. For example, environmental crimes in Brazil have recently caused widespread destruction of natural areas [10], resulting in a significant ecological change [11]. At the same time, the presence of forest and road workers and new properties in the deforested areas triggers a surge in disease transmission, including malaria and arboviruses [11, 12].

In the present review, we analyze current knowledge about outbreaks and reemergence of insect-borne diseases in relation to human migrations and anthropogenic changes in Latin America. We discuss several case studies of interest for public health, including malaria and Chagas disease transmission connected to political crises and migrations, the effects of deforestation, urbanization, and ecotourism on yellow fever and leishmaniasis, and dengue outbreaks in rural areas of the Amazon rainforest.

## Concise history of insect-borne diseases in Latin America

Latin America has been the origin, transit, and destination of millions of international migrants. Besides the historical European migrations since the *conquistadores*, many people have moved to and within Latin America in search of work and education opportunities, as well as refugees fleeing war and persecution [9, 13]. At the same time, Latin America always has been, and still is, afflicted by numerous insect-borne diseases connected to human activities and movements.

Malaria has been present for centuries, with the first reports dating from the 1500s, when *Plasmodium falciparum* was introduced by the African transatlantic slave trade [14]. The introduction of *Plasmodium vivax* into the Latin American region, where it is now the predominant species, happened more than 10,000 years ago [15]. Over the centuries, even with low and unstable transmission rates, malaria has imposed a considerable burden on local populations in the Amazon region [16]. Despite the many efforts made by public health authorities in vector control, medications, and antimalarial drugs, resulting in a substantial decrease in incidence [16], malaria is still endemic in 18 Latin American countries [17] and a reaffirmed risk particularly in Venezuela [18].

American trypanosomiasis (i.e., Chagas disease) was first described in Latin America in the 1900s [19]; however, it was already endemic in the area when the first humans arrived [20]. Chagas disease is caused by *Trypanosoma cruzi*, a protozoan parasite transmitted to humans and animals by the ~154 extant species of triatomine bugs (Hemiptera: Reduviidae), predominantly belonging to the genera *Panstrongylus*, *Psammolestes*, *Rhodnius*, and *Triatoma* [21–23]. Even though the incidence of Chagas disease has decreased over the years in some countries (e.g., Argentina –54.68%, Chile –50.95%, Uruguay –49.95%) thanks to tremendous efforts to eradicate the vectors through home restorations and the use of synthetic insecticides [24], it is still considered a major threat to public health.

Yellow fever arrived in Latin America from Africa with the slave trade during the 1600s [25]. Since then, many outbreaks have occurred, especially in Brazil, with more than 4000 deaths in Rio de Janeiro in the 1850s and the massive 1928–1929 urban epidemic in the same city [26]. Fortunately, the availability of a well-known and affordable vaccine coupled with intense vector control mostly reduced yellow fever to a disease of exclusive sylvatic transmission.

Leishmaniasis, caused by flagellated protozoa of the genus *Leishmania* spp., is transmitted by over 90 phlebotomine sand fly species. The disease manifests in three clinical forms: visceral, the most fatal and occurring especially in Brazil; cutaneous, the most common worldwide; mucocutaneous, having higher incidence among Latin American countries (e.g., Bolivia, Brazil, Peru) [27]. In Latin America, the first autochthonous skin and mucosal manifestations were described in 1909, whereas visceral leishmaniasis has been established since 1934 [28]. While species causing cutaneous leishmaniasis are native to the Neotropical region, genetic data indicate that *Leishmania infantum*, the agent of the visceral form, was introduced to South America by European conquistadors through their dogs [29].

Sporadic cases consistent with the then unknown dengue fever have been reported in Latin America since the 1700s. In the 1800s, a lingering dengue-like outbreak (historical records suggest that it may have been caused by the chikungunya virus brought to America by the African slave trade [30]) affected several countries in Latin America. By 1943–1944, when the dengue virus was first isolated, it had already spread throughout and was endemic in large portions of Latin America [31]. Currently, all Latin American countries are experiencing the highest recorded dengue cases in their history [32, 33].

**Fig. 1:**
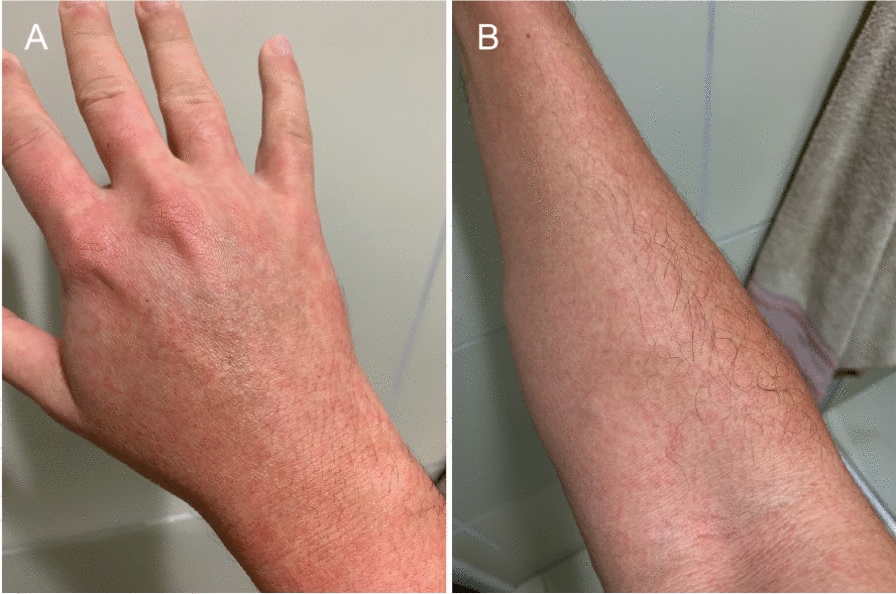
Skin cutaneous rash on the hand (**A**) and forearm (**B**) of a patient from Brazil with acute chikungunya fever. The patient also presented with fever, fatigue, and polyarthritis (photo credit: Filipe Dantas-Torres)

Chikungunya virus was first isolated in 1953 in Tanzania, but it had probably been circulating in Africa and Asia for hundreds of years. In 2005, it spread into the Indian subcontinent and, afterward, the Western Hemisphere. Chikungunya reached Brazil in 2014, making the state the epicenter of American epidemics, with 1,659,167 cases in just two years [34]. Chikungunya is characterized by acute infection (Fig. 1) followed by chronic manifestations such as debilitating musculoskeletal stiffness, joint pain, chronic fatigue, and depression [35, 36]. Asymptomatic cases, which represent 15%, are mainly responsible for the virus spreading among the continents [37]. In November 2023, the first vaccine was approved by the US Food and Drug Administration for adults  18 years of age and older [38], and several other candidates are under development.

More recently, the Zika virus has also been reported in Brazil [39]. The most accepted theory is that the virus circulating in French Polynesia was introduced in Rio de Janeiro during a sports event in 2015 [40]. Then, it rapidly spread through neighboring countries, reaching North America in 2016 [41]. More than 700,000 cases were reported in Latin America from 2015 to 2017, but the incidence has been projected to decrease due to high levels of herd immunity in the population [42].

## Political crisis and malaria transmission in Venezuela

A political, economic, and humanitarian crisis in Venezuela in the early 2010s has resulted in large-scale migration. The decline in oil prices, economic mismanagement, political instability, and international sanctions, often due to human rights violations and the destruction of democratic institutions, led to widespread poverty, food insecurity, hyperinflation, and the collapse of the country’s infrastructure [43]. Therefore, millions of people have fled Venezuela in search of better socioeconomic and living conditions, with many seeking refuge in neighboring Latin American countries [13].

The mass migration out of Venezuela had major public health implications. Millions of people marched through the Amazon jungle to reach the Colombian and Brazilian borders, where they were exposed to highly anthropophilic mosquito vectors of malaria, such as *Anopheles darlingi* Root (Diptera: Culicidae). Furthermore, substandard and overcrowded settlements in border areas facilitated the contact between migrant populations and other *Anopheles* spp. vectors. Then, inadequate mosquito control and deficient health systems (eg.., antimalarial drugs and diagnostic supplies were not sold under medical prescription nor provided by the Venezuelan Government, shortages of indoor and outdoor insecticides for control campaigns) made the migrants particularly vulnerable to malaria [44]. As a result, there was a 1200% increase in cases of malaria in Venezuela compared to the mean number in the early 2000s [18].

The Venezuelan migration crisis has also resulted in a major increase in malaria [45, 46] and other arboviral disease [45] in neighboring countries. Only the Venezuelan government failed to control malaria to such an extent, whereas in Brazil and Colombia, the morbidity, mortality, and economic burden associated with this endemic disease was reduced by more than 50% [44]. This relapse might hinder the global strategy for malaria elimination from the region by at least 90% before 2030 [47] and is a downgrade toward the progress made between 2000 and 2015 [48]. A contribution may come from vaccination. The vaccine RTS,S/AS01, approved by the World Health Organization (WHO) in 2021 after 60 years of development [49], and the more recent R21/Matrix-M, first administered in 2024 in Côte d’Ivoire [50], are recommended for children (from 5 months of age) living in moderate-to-highly affected countries of Africa. Actually, both vaccines protect against malaria caused by *P. falciparum* [51], though *P. vivax* is the predominant species in Latin America, accounting for 72% of cases in 2022 [52]. Therefore, the need for more investment in an effective and safe *P. vivax* vaccine to control the disease is still urgent.

## Migration, inequities, and Chagas disease

Over 150 species of triatomine bugs [23] may transmit *T. cruzi* under experimental conditions, and more than 100 species of mammals are susceptible hosts and reservoirs for its  infection in nature [53]. Vectors and mammal hosts are widespread in Latin America, where Chagas disease occurs endemically in 22 countries. Chagas disease has always been associated with poor living conditions of underserved populations in rural areas in Latin America [54]. However, due to ongoing urbanization processes and mass migration from rural areas to cities, some triatomine bugs have adapted and expanded their distribution range to urban environments by exploiting inequitable conditions in the form of overcrowded and substandard housing conditions [55]. Urban infestations, which have been increasing from the 1990s up to now, and especially in the last three decades, are linked to at least 18 species of triatomine bugs having different degrees of adaptation to the urban environment [56].

In addition, human migration from endemic to nonendemic regions in search of improved living standards has altered the global distribution of Chagas disease [57]. Current estimates point to more than 300,000 imported cases in the USA, 80,000 in Europe, 5000 in Canada, 3000 in Japan, and 1500 in Australia [58]. Latin American countries have been introducing screening on blood units for transfusions searching for microorganisms with proven blood transmission, including *T. cruzi*, since the 1990s [59]. Conversely, most developed countries were not prepared to identify and respond to the challenges posed by Chagas disease. For example, blood banks in Europe and North America have only recently included *T. cruzi* in the list of pathogens routinely tested to screen the millions of blood and organ donors [60, 61].

Another layer of complexity to the already multifaceted epidemiology of Chagas disease transmission is added by acute outbreaks through oral transmission [62]. For example, triatomine bugs are naturally found on açaí palm trees [*Euterpe oleracea* Mart. (Arecales: Arecaceae)], and their crushed berries and juices are traditionally consumed in the Amazon region. Oral transmission occurs when *T. cruzi*-infected triatomine bugs or their feces are unintentionally collected and processed alongside the açaí berries or other contaminated food (e.g., sugarcane, guava juices) [63–65]. Upon ingestion, hosts develop highly symptomatic Chagas disease with an increased mortality rate when compared to infections transmitted by vector bite and defecation [66]. From 2011 to 2020, 2668 cases of acute Chagas disease were reported by health authorities in Brazil alone [67]. Nevertheless, the burden of the disease in other Latin American countries may be underestimated due to underdiagnosing and underreporting [68].

Chagas disease is still a significant public health challenge, which requires the development and implementation of preventative and curative strategies in endemic and nonendemic regions [55, 69]. In this framework, the combined deployment of chemical and non-chemical vector control strategies and the improvement of housing conditions may help reduce insect vector populations in the (peri)domestic environment [70]. The management of domestic populations of triatomines that are also found in sylvatic environments is challenging, as the possibilities of re-infestation after the treatments are, unfortunately, higher [71]. To limit the insurgence of orally transmitted Chagas disease in areas at risk, it is crucial to inform fruit producers and sellers about basic precautionary procedures to prevent *T. cruzi* contamination during storage and processing, as well as develop education campaigns to inform locals and tourists about the hazard posed by food consumption in certain zones [65].

## Yellow fever and travelers

Yellow fever is considered endemic in the Amazon rainforest and parts of some countries (e.g., Peru, Colombia, Venezuela, Brazil), and its jungle transmission is mainly maintained by *Haemagogus* spp. and *Sabethes* spp. mosquitoes (Diptera: Culicidae) [72]. Yellow fever transmission in sylvatic cycles occurs when such mosquitoes blood feed on non-human primates [73]. However, humans living or traveling through the Amazonia or along the border between forested and peri-urban or rural areas are at increased risk of being bitten by infected mosquitoes, especially during the warmer months of the year when they are more abundant and travelers are engaging in outdoor daylight activities in the region [74, 75].

**Fig. 2:**
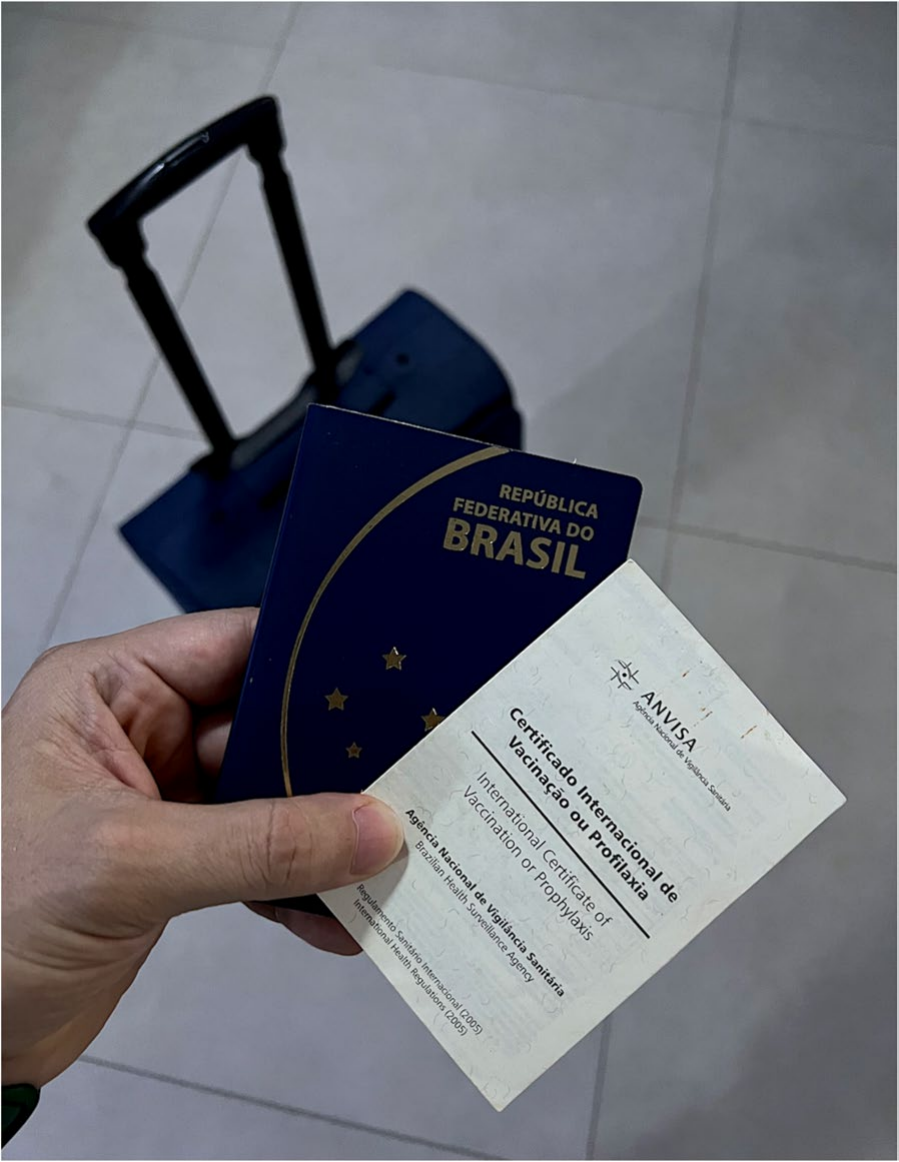
International certificate of yellow fever vaccination. The certificate validity used to be 10 years, but now it is lifelong (photo credit: Filipe Dantas-Torres)

The implementation of mosquito control strategies in the forest is unrealistic due to its inherent characteristics (e.g., dense vegetation, high humidity, abundant backwater, vast land area). Therefore, the response from the public health authorities to deal with the threat of yellow fever transmission in the region has been primarily focused on vaccination as a preventive measure [76]. A cost-effective vaccine, named 17D, has been available since 1937 [77]. According to the WHO, a single dose provides lifelong lasting immunity for most people [78]. The WHO also provides a listing of countries at risk of yellow fever transmission and those requiring proof of vaccination (Fig. 2) against it [79]. In addition, it offers an interactive map with information on yellow fever transmission and vaccination requirements for the Americas [80].

**Fig. 3:**
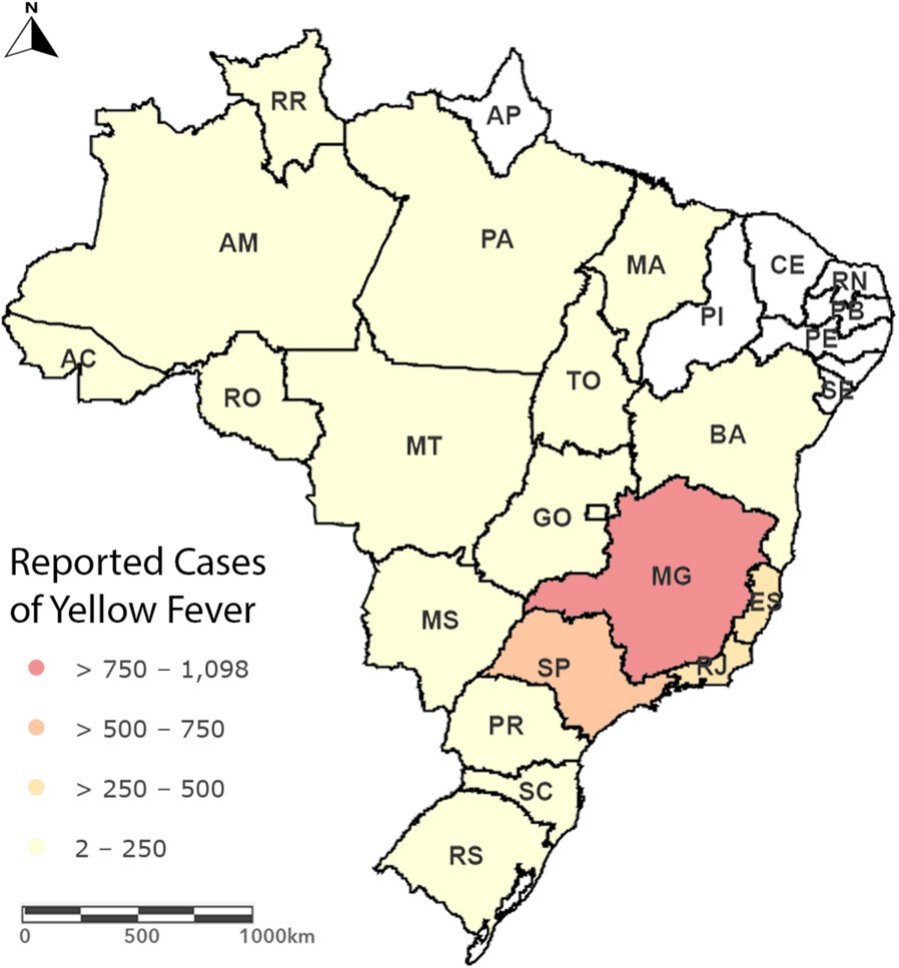
Reported human cases of yellow fever in Brazil from 1994 to 2023. The southeast region reported the highest number of cases, likely due to tourists encountering vector mosquitoes that are endemic to the Atlantic Forest. The figure was produced using ArcGIS 10.2 (Esri, Redlands, CA)

Occasionally, *Ae. aegypti* and *Ae. albopictus* are responsible for the transmission in urban and peri-urban environments [81], as in the cases of urban epidemics in Brazil back in 1935–1940 [25]. More recently, an urban outbreak of yellow fever took place in Asunción, the capital city of Paraguay, but *Ae. aegypti* control and a massive vaccination effort contained the damage [82]. The yellow fever virus also reemerged in central-western Brazil in 2014, and then spread to eastern and southern regions of the country [73, 83, 84]. From 1994 to 2023 (Fig. 3), 2768 reported cases and 1010 deaths were recorded [85], with most of the infected individuals being unvaccinated people.

The recent yellow fever outbreak in Brazil exemplifies the need to improve preparedness and response. The detection of *Ae. albopictus* specimens infected with yellow fever in a transmission hotspot area in Minas Gerais, Brazil (part of the Atlantic Forest biome) [86] elevated their status as a potential bridge species from a sylvatic to an urban transmission cycle [87, 88]. Due to increasing deforestation and urbanization of natural areas, the risk of urban transmission of yellow fever mediated by *Ae. aegypti* poses a substantial threat to public health.

## Deforestation, urbanization, ecotourism, and leishmaniasis

**Fig. 4:**
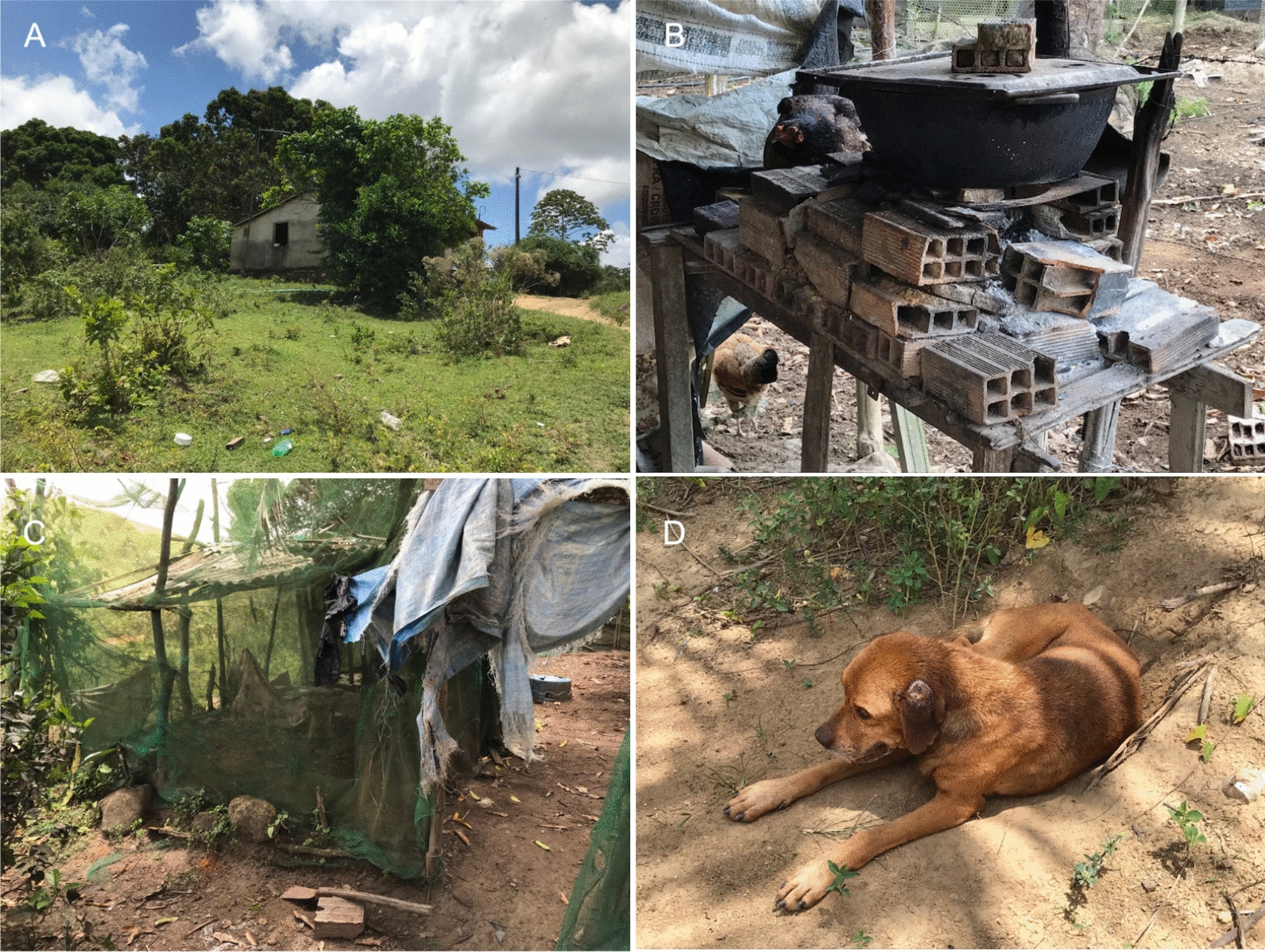
Poor housing and environmental conditions (**A**) in an area where cutaneous leishmaniasis by *Leishmania braziliensis* is endemic. A precarious oven (**B**), a chicken pen (**C**), and a dog with an ulcer on the ear (**D**). Dogs in this area are also frequently infected by *L. braziliensis* (photo credit: Filipe Dantas-Torres)

Leishmaniasis is a growing global public health threat caused by *Leishmania* spp. protozoa, which are transmitted by several species of phlebotomine sand flies [89]. In Latin America, leishmaniasis has been historically associated with natural areas, and cases were rarely reported in urban contexts [90]. However, during the past decades, the disease has also been spreading to urban areas [91]. Factors influencing this urbanization process of leishmaniasis (especially the visceral form of the disease) include the capacity of vector sand flies to thrive in urban settings [92], deforestation [93], use of former forest areas for infrastructure development (e.g., road construction) [94], and movement of human and canine populations from rural and urban areas [95]. Still, the burden of leishmaniasis remains heavier in underserved rural environments in Latin America, being strongly linked to poor housing conditions (Fig. 4) and high levels of exposure to sand flies.

A minor trend in leishmaniasis transmission has been reported by many studies in relation to travelers (e.g., adventure travelers, military personnel, researchers) [89, 96, 97]. The interest in ecotourism and business trips has exposed visitors from many regions of the world to infectious sand fly bites. For this reason, Latin America is now considered a critical transmission zone of leishmaniasis among tourists who visit natural areas (e.g., rivers, waterfalls, caves) bearing high risk of infection [98–100], especially of the cutaneous form [101].

Currently, there are no licensed vaccines for the prevention of human leishmaniasis, and since this neglected tropical disease primarily concerns low- and middle-income countries, commercial developers are less interested in them [102]. One viable solution is transmission prevention, relying on sand fly bite avoidance through personal care measures (e.g., repellents on clothes and exposed skin) and the use of insecticide-treated nets.

## Dengue in the Amazon

*Aedes albopictus* and *Ae. aegypti* are commonly identified as the primary vectors of arboviruses of public health importance [103, 104]. *Aedes aegypti* is a highly competent vector of chikungunya, dengue, yellow fever, and Zika, causing single and multiple infections, with Brazilian mosquito populations being particularly susceptible to dengue infections [105, 106]. In Brazil, *Ae. albopictus* is commonly considered a vector of relatively minor significance [107], although it has been present since 1986 [108] and is currently spread in 24 out of 27 states [109]. Those *Aedes* species greatly benefit from urbanization due to abundant blood sources, especially in densely populated areas, fewer natural predators, and widely available human-made aquatic habitats [6].

Over the last 30 years, the dengue burden has progressed worldwide at the same pace as socioeconomic development, climate warming, and human population movements [110]. In remote and agricultural areas of Brazil, the deforestation of natural areas for the creation of rural developments has led to a major increase in the incidence of dengue [111, 112]. Because of the creation of rural developments, not only did the human population density increase due to the migration of workers (both to work on the rural developments and provide infrastructure and leisure), but new dengue serotypes were introduced into naïve human populations, including local indigenous natives [113]. Furthermore, migrants working in mining and rural developments were more severely affected due to overcrowded and substandard living conditions, increased exposure to mosquito vectors, and limited access to health care [114]. These factors are major drivers for the increased risk of dengue transmission and spread [110, 115, 116]. As a result, the highest dengue incidence in Brazil was reported in rural areas. For instance, in 2022, the state of Acre experienced 1594 cases of dengue per 100,000 people, followed by 770 dengue cases in Goiás, 592 in Mato Grosso, and 534 in Tocantins [117].

In the first weeks of 2020, despite the ongoing COVID-19 pandemic and limited human population movements, Brazil reported an increased number of dengue cases [118], whereas a few months later, the country noted an abrupt decrease in dengue notifications, probably because of social isolation or underreporting [119]. Therefore, the above-listed numbers might even be underestimated.

Data obtained from the passive surveillance system in Neiva, Colombia showed a significant occurrence of arboviral diseases (i.e., more than 6% of the population), mainly dengue (followed by Zika and chikungunya), among populations internally displaced by armed conflicts or persecution [120]. In these situations, political commitment is fundamental to decreasing the number of populations considered internally displaced in the country and including them in an equitable health system with appropriate health coverage [120].

Currently, two dengue vaccines against the four virus serotypes are commercially available: one (CYD-TDV) is only recommended for already infected children and adults (ages 9–45 years) living in endemic areas, and the other (TAK-003) as a form of prevention, suitable for children and adults (ages 4–60 years), with or without previous dengue infections. CYD-TDV, approved in Brazil, Mexico, El Salvador, Costa Rica, Paraguay, Peru, Guatemala, and other countries outside Latin America, appears to increase the risk of more severe symptoms if injected in naïve patients [45]. This adverse outcome reduces the total number of individuals who can be vaccinated in endemic countries, thus negatively impacting the implementation of solid preventive measures [121]. Since September 2023, TAK-003 has been officially recommended by the WHO for the routine immunization programs of children (ages 6–16 years) in settings with high dengue transmission intensity [122]. Despite the WHO prequalification, the vaccine is currently only available in Brazil, which is the first country in the world to implement dengue vaccination at the national level [123, 124].

## Future challenges

Insect-borne diseases continue to significantly threaten public health in Latin America. The complex interaction among vector ecology, human behavior, and environmental and socioeconomic conditions are relevant drivers for the risk of pathogen transmission in the region. In this framework, human migration historically played a crucial role in the spread of insect-borne diseases in Latin America. Both domestic and international migrations can bring susceptible human populations into areas of active transmission, as well as introduce vectors and pathogens into previously free regions. Migrants, refugees, and displaced populations are particularly vulnerable to insect-borne diseases due to their often-limited access to health care and inadequate living conditions.

For diseases with already available and recommended vaccines, such as dengue, chikungunya, and yellow fever, the implementation of proper vaccination campaigns still needs to be improved in some Latin American countries. For instance, despite the declared efficacy of the yellow fever vaccine under laboratory conditions, its application has been sometimes hindered by logistical challenges in production and distribution, along with issues concerning public adherence to the initiative [125–127]. Importantly, the development of vaccines and drugs for insect-borne neglected diseases, such as  human leishmaniasis and Zika, should be treated as a priority by governmental and nongovernmental organizations, as well as by the pharmaceutical industry.

Particularly in resource-limited regions, traditional surveillance and control methods remain essential for informing public health authorities on hot spot areas for insect-borne disease transmission and subsequent response through control interventions. In most situations, vector control remains a cardinal component of outbreak response, but the increasing levels of insecticide resistance in mosquitoes [128] and triatomine bugs [129] pose a significant challenge to the implementation of effective, environmentally friendly, and long-term sustainable control strategies. Regrettably, vector control is not applicable to fully avoid oral transmission of Chagas disease and is unfeasible in the case of outbreaks in challenging environments such as forests.

In the current scenario of intense migrations coupled with severe anthropogenic changes, successful control strategies should follow the integrated vector management (IVM) framework by combining all the available resources and tools to develop effective and sustainable control interventions. Technology can also be a powerful tool if implemented under the IVM framework [130]. Geographic Information Systems (GIS) and statistical and simulation models (including the forecast of disease transmission and projection of the effectiveness of control interventions) provide good opportunities for enhancing disease and vector surveillance and the evaluation of the effectiveness of control strategies [131, 132]. The development and implementation of sustainable control strategies remains a major challenge in resource-limited countries. For instance, a single injectable dose of fluralaner (isoxazoline) in dogs has been proven efficacious in managing tick and flea infestations for one year [133, 134]. This strategy could be further explored to control diseases such as leishmaniasis by *L. infantum*, considering the role of dogs as reservoirs of this parasite.

At the same time, the lack of robust and reliable surveillance systems, as well as their integration with data analysis capabilities to allow the development of scientific-driven interventions and the implementation of early warning systems, are major obstacles for public health authorities to adequately respond to outbreaks and track disease transmission. Interdisciplinary collaboration and data sharing across different sectors and stakeholders involved in research and control, including public health authorities, scientists, and local communities, should also be one of the main goals.

Surveillance systems are especially important to respond to insect-borne disease transmission in urban areas. Urbanization creates a wide range of habitats for the proliferation of vector species, while also increasing human population density and, therefore, making the contact between insect vectors and human hosts more frequent. The situation is even worse for vulnerable populations such as migrants, refugees, and underserved communities who often must endure substandard living conditions and limited access to health care, thus being more exposed to insect vectors and having poorer health outcomes when infected with pathogens. Promoting the engagement of communities, public health authorities, and stakeholders in addressing the social and economic determinants of health that contribute to insect-borne diseases is crucial to face the future challenges of reducing this burden in Latin America.

## Conclusions

The outbreaks or reemergence of insect-borne diseases that have been historically afflicting Latin America or were more recently described and have lately expanded are often related to human migrations and anthropogenic changes such as urbanization, deforestation, and ecotourism. Even the Oropouche fever, an alarming arboviral disease historically maintained in sylvatic cycles in the Amazon basin only and now threatening Brazil, Peru, Bolivia, and Colombia [135], may be linked to human mobility and the consequent population density increase [136]. The complex interactions among vectors, pathogens, humans, and environmental and climate changes are not fully understandable or predictable. Addressing the social, economic, and political determinants of health that impact the burden of insect-borne diseases in Latin America is essential for reducing their transmission, particularly for the most vulnerable populations. The key strategies to lower their incidence in Latin America include a higher vaccination coverage for diseases with already available and recommended formulations (e.g., dengue, chikungunya, yellow fever), as well as the implementation of refined vector surveillance systems and control programs. Improving the basic sanitation, potable water supply, and housing conditions in the most affected areas is just as important. Finally, social media (e.g., X, previously Twitter [137]) and social messaging apps (e.g., WhatsApp [138]) may also play a key role in increasing community engagement and outreach of control activities to mitigate insect-borne diseases in Latin America.

## Data Availability

No datasets were generated or analysed during the current study.

## Abbreviations

GIS: Geographic Information Systems
IVM: Integrated vector management
WHO: World Health Organization
